# Updates on cardiovascular outcome trials in diabetes

**DOI:** 10.1186/s12933-017-0610-y

**Published:** 2017-10-11

**Authors:** Oliver Schnell, Lars Rydén, Eberhard Standl, Antonio Ceriello, on behalf of the D&CVD EASD Study Group

**Affiliations:** 1 Forschergruppe Diabetes e. V., Ingolstaedter Landstraße 1, Neuherberg, 85764 Munich, Germany; 2 Cardiology Unit, Department of Medicine K2, Karolinska Institute, 171 76 Stockholm, Sweden; 3 Institut d’Investigacions Biomèdiques August Pi i Sunyer (IDIBAPS) and Centro de Investigación, Biomedica en Red de Diabetes y Enfermedades Metabólicas Asociadas (CIBERDEM), Barcelona, Spain; 4 Department of Cardiovascular and Metabolic Diseases, IRCCS Multimedica Sesto San Giovanni, Via Milanese 300, 20099 Milan, Italy

**Keywords:** Cardiovascular risk, Diabetes, CVOT, DEVOTE, CANVAS program, EXSCEL, ACE, Heart

## Abstract

In 2008 the Food and Drug Administration introduced a guidance for industry that requires the investigation of cardiovascular outcomes of glucose-lowering medications. Since then, an increasing number of cardiovascular outcome trials have been completed in diabetes patients with high cardiovascular risk for members of the SGLT-2 and DPP4 inhibitors and GLP-1 receptor agonist classes. The trials confirmed cardiovascular safety for all tested anti-hyperglycaemic drugs and, in addition empagliflozin, semaglutide and liraglutide could even reduce cardiovascular risk. The present review summarizes the results of the DEVOTE, CANVAS, EXSCEL and ACE trials that tested cardiovascular safety of Insulin degludec, canagliflozin, once-weekly exenatide and acarbose and were published in 2017. We provide context on these results by comparing them with earlier trials of glucose-lowering drugs and give an outlook on what to expect in coming years.

## Background

Patients with diabetes have a much higher risk of cardiovascular (CV) disease (CVD) than individuals without diabetes [1]. This up to 50% increased risk of CV-related death is one of the major causes of mortality [2]. Studies could show, that good glycaemic control can positively influence the long-term development of CVD and mortality [3, 4]. Thus, CV safety and benefits of glucose-lowering medications have been the focus of recent studies. Accordingly, the Food and Drug Administration (FDA) and the European Medicines Agency (EMA) presented a guidance for the approval of glucose-lowering medications in 2008 and 2012, respectively [5, 6]. This guidance requires the assessment of CV safety: if the pre-marketing application data revealed a hazard ratio (HR) with an upper 95% confidence interval (CI) between 1.3 and 1.8, a post-marketing trial will generally be necessary to demonstrate an upper 95% CI of < 1.3. Does the pre-marketing clinical data already demonstrate an upper 95% CI of < 1.3, the post-marketing trial can be neglected [5, 7]. Since issuing the abovementioned guidelines, several major CV outcome trials (CVOTs) had been completed until 2016. These included glucose-lowering medications of the DPP-4 (3 studies: SAVOR-TIMI53, EXAMINE and TECOS) and SGLT-2 inhibitor (1 study: EMPA-REG OUTCOME) as well as GLP-1 receptor agonist (RA; 3 studies: ELIXA, LEADER and SUSTAIN6) classes [8]. Also Insulin glargine and Insulin degludec had been subjected to be tested for CV safety [9–11].

The previously published CVOTs determined safety of the DPP-4 inhibitors saxagliptin, alogliptin and sitagliptin as well as the GLP-1 RA lixisenatide with regard to CV outcomes [12–15]. Additionally, LEADER, SUSTAIN6 and EMPA-REG OUTCOME could show the capability of liraglutide, semaglutide and empagliflozin to reduce CV outcomes in diabetes patients with high CV risk [16–18]. The D&CVD EASD Study Group recently gave an overview of these CVOTs, and discussed future perspectives for the treatment of patient with diabetes (Tables 1, 2 and 3; [8]).

**Table 1:** Basic characteristics of CVOTs started after 2008 FDA regulation

	Study status	Drug	Drug class	Intervention	Primary outcome	N	Follow-up (years)	Start and estimated end date	Clinicaltrials.gov ID
SAVOR-TIMI53	Completed	Saxagliptin	DPP-4 inhibitor	Addition of saxagliptin vs. placebo to usual diabetes care	CV death, MI, or stroke	18,206	2.1	05.2010–05.2013	NCT01107886
EXAMINE	Completed	Alogliptin	DPP-4 inhibitor	Addition of alogliptin vs. placebo to usual diabetes care	CV death, MI, or stroke	5380	1.5	10.2009–06.2013	NCT00968708
TECOS	Completed	Sitagliptin	DPP-4 inhibitor	Sitagliptin vs. placebo	CV death, MI, UA, or stroke	14,724	3	12.2008–03.2015	NCT00790205
ELIXA	Completed	Lixisenatide	GLP-1 receptor agonist	Lixisenatide vs. placebo	CV death, MI, UA, or stroke	6076	2.1	06.2010–02.2015	NCT01147250
EMPA-REG OUTCOME	Completed	Empagliflozin	SGLT-2 inhibitor	Empagliflozin 10 mg vs. empagliflozin 25 mg vs. placebo	CV death, MI, or stroke	7000	3.1	07.2010–04.2015	NCT01131676
LEADER	Completed	Liraglutide	GLP-1 receptor agonist	Liraglutide vs. placebo	CV death, MI, or stroke	9340	3.8	08.2010–12.2015	NCT01179048
SUSTAIN-6	Completed	Semaglutide	GLP-1 receptor agonist	Semaglutide 0.5 mg vs. semaglutide 1.0 mg vs. placebo	CV death, MI, or stroke	3299	1.9	02.2013–01.2016	NCT01720446
EXSCEL	Completed	Exenatide	GLP-1 receptor agonist	Exenatide once-weekly vs. placebo	CV death, MI, or stroke	14,752	3.2	06.2010–04.2017	NCT01144338
CAROLINA	Ongoing, not recruiting	Linagliptin	DPP-4 inhibitor	Linagliptin vs. sulfonylureas vs. placebo	CV death, MI, UA, or stroke	6000	–	10.2010–03.2019	NCT01243424
REWIND	Ongoing, not recruiting	Dulaglutide	GLP-1 receptor agonist	Dulaglutide vs. placebo	CV death, MI, or stroke	9622	–	07.2011–07.2018	NCT01394952
ITCA650	Completed	Exenatide in DUROS	GLP-1 receptor agonist	ITCA 650 (exenatide in DUROS) vs. placebo	CV death, MI, UA, or stroke	4000	–	03.2013–03.2016	NCT01455896
DECLARE-TIMI	Ongoing, not recruiting	Dapagliflozin	SGLT-2 inhibitor	Dapagliflozin 10 mg vs. placebo	CV death, MI, or stroke	17,276	–	01.2013–04.2019	NCT01730534
CARMELINA	Ongoing, not recruiting	Linagliptin	DPP-4 inhibitor	Linagliptin vs. placebo	CV death, MI, UA, or stroke	8000	–	07.2013–12.2017	NCT01897532
DEVOTE	Completed	Insulin degludec	Basal insulins	Insulin degludec vs. insulin glargine	CV death, MI, or stroke	7637	1.9	10.2013–10.2016	NCT01959529
MK-3102	Terminated	MK-3102	DPP-4 inhibitor	MK-3102 vs. placebo	CV death, MI, UA, or stroke	4202	–	10.2012–03.2017	NCT01703208
VERTIS	Ongoing, not recruiting	Ertugliflozin	SGLT-2 inhibitor	Ertugliflozin 5 mg vs. ertugliflozin 15 mg vs. placebo	CV death, MI, or stroke	3900	–	11.2013–10.2019	NCT01986881
CANVAS program	Completed	Canagliflozin	SGLT-2 inhibitor	Canagliflozin 100 mg vs. canagliflozin 300 mg vs. placebo	CV death, MI or stroke	10,142	1.5	12.2009–02.2017	NCT01032629
Albiglutide trial	Ongoing, not recruiting	Albiglutide	GLP-1 receptor agonist	Albiglutide 30 mg vs. albiglutide 50 mg vs. placebo	CV death, MI or stroke	9400	–	07.2015–02.2018	NCT02465515
ACE	Completed	Acarbose	α-Glucosidase inhibitor	Acarbose vs. placebo	CV death, MI or stroke	6522	5.0	02.2009–04.2017	NCT00829660

**Table 2:** Inclusion criteria of patients enrolled in CVOTs referred to in the text

	Age	Diabetes type	HbA1c levels	Cardiovascular status	Prior anti hyperglycaemic treatment	BMI (Kg/m^2^)
SAVOR-TIMI53	≥ 40	T2DM	≥ 6.5%	CVD or high CV risk	AHA	31.1
EXAMINE	≥ 18	T2DM	(6.5, 11.0%)	ACS (15, 90) days before	AHA	28.7
TECOS	≥ 50	T2DM	(6.5, 8.0%)	pre-existing CVD	AHA	30.2
ELIXA	≥ 30	T2DM	≥ 7.0%	ACS min. 180 days before	AHA	30.2
EMPA-REG OUTCOME	≥ 18	T2DM	(7.0, 10.0%)	Pre-existing CVD	Drug naive or AHA	≤ 45
LEADER	≥ 50	T2DM	≥ 7.0%	Pre-existing CVD/cerebrovascular disease/vascular disease/renal or heart failure at ≥ 50 or CV risk at ≥ 60	Drug naive or AHA	32.5
SUSTAIN6	≥ 50	T2DM	≥ 7.0%	Pre-existing CVD at ≥ 50 OR pre-CVD at ≥ 60	Drug naive or AHA	31.1
EXSCEL	≥ 18	T2DM	6.5–10.0%	73.1% with previous CVD	Specific AHA	–
CAROLINA	≥ 40 ≤ 85	T2DM	(6.5, 7.5–8.5%)	CVD or specified diabetes end-organ damage or age ≥ 70 years or ≥ 2 specified CV risk factors	–	≤ 45
REWIND	≥ 50	T2DM	≤ 9.5%	Pre-existing vascular disease or ≥ CV risk factors	AHA	–
ITCA650	≥ 40	T2DM	≥ 6.5%	Pre-existing coronary, cerebrovascular or peripheral artery disease	–	–
DECLARE-TIMI	≥ 40	T2DM	–	High risk CV events	–	–
CARMELINA	≥ 18	T2DM	(6.5, 10.0%)	High risk CV events	Drug naive or specific AHA	≤ 45
DEVOTE	≥ 50	T2DM	≤ 7.0%	CVD or renal disease or ≥ 60 CV risk	Specific AHA	–
MK-3102	≥ 40	T2DM	(6.5, 10.0%)	Pre-existing vascular disease	–	–
VERTIS	≥ 40	T2DM	(7.0, 10.5%)	Pre-existing vascular disease	Drug naive or AHA	≥ 18
CANVAS program	≥ 40	T2DM	(7.0, 10.5%)	Pre-existing CVD or high CV risk	Drug naive or AHA	–
Albiglutide trial	≥ 40	T2DM	> 7.0%	CVD	–	–
ACE	≥ 65	Prediabetes	5.9%	CV event within the last 3 month	Drug naive	25

**Table 3:** Concomitant medication at baseline in CVOTs referred to in the text

Concomitant medication @baseline	Antihyperglycaemic medication N (%)			CV treatment N (%)					
	Insulin	Metformin	Sulphonyl-urea	Aspirin	Statins	Antiplatelet/anticoagulant	Beta-blocker	ACEI/ARB	Other anti-hypertensives
SAVOR-TIMI53	6757 (40.9)	11,094 (67.4)	6332 (38.5)	12,390 (75.2)	12,892 (78.3)	13,386 (81.3)	10,117 (61.4)	12,935 (78.5)	6730 (40.9)
EXAMINE	1605 (29.8)	3562 (66.2)	2503 (69.9)	4881 (90.7)	4866 (90.4)	5232 (97.2)	4411 (81.9)	4411 (81.9)	1197 (22.2)
TECOS	3408 (23.2)	11,966 (81.6)	6645 (45.3)	11,518 (78.5)	11,719 (79.9)	3167 (21.7)	9322 (63.5)	11,555 (78.8)	4961 (33.8)
ELIXA	2292 (37.8)	3834 (63.2)	1863 (30.7)	5726 (94.4)	5621 (92.6)	480 (7.9)	5119 (84.4)	5151 (84.9)	1327 (21.9)
EMPA-REG OUTCOME	2374 (34.0)^a^	3933 (55.9)^a^	1383 (19.6)	5990 (85)	5387 (77)	–	4537 (64)	5651 (80)	2114 (30)
LEADER	4159 (45.0)^a^	7136 (76.4)	4721 (50)	5874 (63)	6729 (72)	6322 (67.7)	5173 (55.4)	7731 (83)	920 (9.85)
SUSTAIN 6	1 913 (58.0)	2414 (73.2)	1410 (42.8)	2108 (63.9)	2399 (72.8)	406 (12.3)	1894 (57.4)	2753 (83.5)	258 (7.8)
EXSCEL	6836 (46.3)	11,295 (76.6)	5401 (36.6)	9380 (63.6)	10,845 (73.5)	10,835 (73.5)	8211 (55.7)	11,788 (79.9)	
DEVOTE	6409 (83.9)	4564 (59.8)	2229 (29.2)	4764 (62.4)	5972 (78.2)	1599 (20.9)	4370 (57.2)	6182 (80.9)	2458 (32.2)
CANVAS program	5095 (50.2)	7825 (77.2)	4361 (43)	–	7599 (74.9)	7466 (73.6)	5421 (53.5)	–	–
ACE	–	–	–	6131 (94)	6066 (93)	6384 (98)	4301 (66)	3839 (59)	–

^a^Both mono and dual therapy

Here we present an update of this publication, including the most recent CVOTs on GLP-1 RAs, SGLT-2 inhibitors, Insulin degludec and acarbose and discuss their implications for the medication of type 2 diabetes mellitus (T2DM) patients.

## Summary of recently completed CVOTs

In the past year, several CVOTs were completed, among them DEVOTE, ITCA650 (FREEDOM-CVO), the CANVAS program and very recently the EXSCEL and ACE trials. Four of these studies represent common drug classes for glucose-lowering medication in diabetes: basal insulin, GLP-1 RAs and SGLT-2 inhibitors. One study investigating the CVO of the DPP-4 inhibitor MK-3102 was terminated in March 2017. The DEVOTE study compared the CVO of the ultra-long-acting Insulin degludec with Insulin glargine U100 in T2DM patients with a high risk of CV events. The trial was designed to continue until the occurrence of at least 633 primary outcome events [10, 19]. The CANVAS program comprises two sister trials that were combined to increase statistical power to assess CV safety and efficacy of canagliflozin. The original CANVAS design was adjusted after unblinding the trial for sub-group analysis in 2012. Because of the observed interim CVO results, a second separate study (CANVAS-R) was initiated. The inclusion criteria of CANVAS and CANVAS-R were almost identical. In addition to the primary MACE outcome the separate primary objective of CANVAS-R is progression of albuminuria [20, 21]. Two recently completed studies evaluated the CV risk of exenatide: EXSCEL and FREEDOM-CVO. EXSCEL, the largest GLP-1 RA study to date, analysed the influence of a once weekly subcutaneous injection of exenatide whereas in FREEDOM-CVO participants were continuously supplied with exenatide via an implanted mini pump (DUROS^®^). Additionally, the ACE trial investigated whether the α-glucosidase inhibitor acarbose could reduce CV events in a population with established coronary heart disease and newly detected impaired glucose tolerance. This population differed from those investigated in the other CVOTs which included T2DM patients. The original study design planned to investigate a three point MACE, which, due to too few events was extended to a five point MACE. The ACE study had the longest follow-up period of CVOTs summarized in this review (mean of 5 years [22, 23]).

The major results of the completed CVOTs are summarized in the following sections, divided by CV outcomes: Primary MACE composite endpoint, all-cause mortality, myocardial infarction (MI), unstable angina (UA), CV death and heart failure (HF). Subsequently the safety endpoints renal events, pancreatitis, hypoglycaemic episodes and amputations will be reviewed (Table 4).

**Table 4:** Comparison of outcome results from terminated CVOTs in comparison to placebo

	SAVOR-TIMI53 [12, 47]		EXAMINE [13, 48]		TECOS [14]		ELIXA [15]		EMPA-REG OUTCOME [18, 48]		LEADER [49]	
	Class	HR (95% CI)	Class	HR (95% CI)	Class	HR (95% CI)	Class	HR (95% CI)	Class	HR (95% CI)	Class	HR (95% CI)
		p value		p value		p value		p value		p value		p value
Cardiovascular endpoints												
Primary composite MACE	CV death, MI, or stroke	1.00 (0.89–1.12) 0.99	CV death, MI, or stroke	0.96 (≤ 1.16) 0.32	CV death, MI, UA, or stroke	0.98 (0.89–1.08) 0.65	CV death, MI, UA, or stroke	1.02 (0.89–1.17) 0.81	CV death, MI, or stroke	0.86 (0.74–0.99) 0.04^a^	CV death, MI, or stroke	0.87 (0.78–0.97) 0.01
Cardiovascular death	Primary endpoint	1.03 (0.87–1.22) 0.72	Primary endpoint	0.85 (0.66–1.10) 0.212	Primary endpoint	1.03 (0.89–1.19) 0.71	Primary endpoint	0.98 (0.78–1.22) 0.85	Primary endpoint	0.62 (0.49–0.77) < 0.001	Primary endpoint	0.78 (0.66–0.93) 0.007
Myocardial infarction	Primary endpoint	0.95 (0.80–1.12) 0.52	Primary endpoint	1.08 (0.88–1.33) 0.47	Primary endpoint	0.95 (0.81–1.11) 0.49	Primary endpoint	1.03 (0.87–1.22) 0.71	Primary endpoint	0.87 (0.70–1.09) 0.23	Primary endpoint	0.86 (0.73–1.00) 0.046
Stroke	Primary endpoint	1.11 (0.88–1.39) 0.38	Primary endpoint	0.91 (0.55–1.50) 0.71	Primary endpoint	0.97 (0.79–1.19) 0.76	Primary endpoint	1.12 (0.79–1.58) 0.54	Primary endpoint	1.18 (0.89–1.56) 0.26	Primary endpoint	0.86 (0.71–1.06) 0.16
Hospitalization for unstable angina	Secondary endpoint	1.19 (0.89–1.60) 0.24	Secondary endpoint	0.90 (0.60–1.37) 0.632	Primary endpoint	0.90 (0.70–1.16) 0.42	Primary endpoint	1.11 (0.47–2.62) 0.81	Secondary endpoint	0.99 (0.74–1.34) 0.97	Extended primary endpoint	0.98 (0.76–1.26) 0.87
Hospitalization for heart failure	Secondary endpoint	1.27 (1.07–1.51) 0.007	Extended primary endpoint	1.19 (0.90–1.58) 0.220	Secondary endpoint	1.00 (0.83–1.20) 0.98	Secondary endpoint	0.96 (0.75–1.23) 0.75	Secondary endpoint	0.65 (0.50–0.85) 0.002	Extended primary endpoint	0.87 (0.73–1.05) 0.14

	Event rate (%) active group		Event rate (%) active group		Event rate (%) active group		Event rate (%) active group		Event rate (%) active group		Event rate (%) active group	
Primary composite MACE	7.3		11.3		9.6		13.4		10.5		13.0	

	No. (%) p value		No. (%) p value		No. (%) p value		No. (%) p value		No. (%) p value		No. (%) p value	
Non-cardiovascular endpoints												
Renal event	5.8 0.04		0.9 0.88		1.4 –		1.6 –		5.2 –		5.7 0.003	
Acute pancreatitis	0.3 0.77		0.4 0.5		0.3 0.07		0.2 –		0.3^b^ –		0.4 0.44	
Hypoglycemia events	15.3 < 0.001		6.7 0.74		2.2 0.33		16.6 0.14		2.8		2.4 0.02	

	SUSTAIN 6 [50]		EXSCEL [24]		DEVOTE [10]		CANVAS program [21]		ACE [23]	
	Class	HR (95% CI) p value	Class	HR (95% CI) p value	Class	HR (95% CI) p value	Class	HR (95% CI) p value	Class	HR (95% CI) p value
Cardiovascular endpoints										
Primary composite MACE	CV death, MI, or stroke	0.74 (0.58–0.95) 0.02^a^	CV death, MI or stroke	0.91 (0.83–1.00) 0.06^a^	CV death, MI or stroke	0.86 (0.75–0.97) 0.02^a^	CV death, MI or stroke	0.86 (0.75–0.97) 0.02^a^	CV death, MI, stroke, UA and HF	0.98 (0.86–1.11) 0.73
Cardiovascular death	Primary endpoint	0.98 (0.65–1.48) 0.92	Secondary endpoint	0.88 (0.76–1.02)	Primary endpoint	0.87 (0.72–1.06) < 0.001	Primary endpoint	0.87 (0.72–1.06) < 0.001	Secondary endpoint	0.89 (0.71–1.11) 0.29
Myocardial infarction	Primary endpoint	0.74 (0.51–1.08) 0.12	Secondary endpoint	0.97 (0.85–1.10)	Primary endpoint	0.89 (0.73–1.09) < 0.001	Primary endpoint	0.89 (0.73–1.09) < 0.001	Secondary endpoint	1.12 (0.87–1.46) 0.38
Stroke	Primary endpoint	0.61 (0.38–0.99) 0.04	Secondary endpoint	0.85 (0.70–1.03)	Primary endpoint	0.87 (0.69–1.09) < 0.001	Primary endpoint	0.87 (0.69–1.09) < 0.001	Secondary endpoint	0.97 (0.70–1.33) 0.83
Hospitalization for unstable angina	Extended primary endpoint	0.82 (0.47–1.44) 0.49	–	–	–	–	–	–	Secondary endpoint	1.02 (0.82–1.26) 0.87
Hospitalization for heart failure	Extended primary endpoint	1.11 (0.77–1.61) 0.57	Secondary endpoint	0.94 (0.78–1.13)	Secondary endpoint	0.67 (0.52–0.87) < 0.001	Secondary endpoint	0.67 (0.52–0.87) < 0.001	Secondary endpoint	0.89 (0.63–1.24) 0.48

	Event rate (%) active group	Event rate (%) active group	Event rate (%) active group	Event rate (%) active group	Event rate (%) active group
Primary composite MACE	6.6	11.4	8.5	26.9^d^	14.4

	No. (%) p value	No. (%) p value	No. (%) p value	No. (%) p value	No. (%) p value
Non-cardiovascular endpoints					
Renal event	3.9 –	55 (0.7)	3.8 –	19.7^d^ 0.32	–
Acute pancreatitis	0.55 –	26 (0.4)	– –	0.5^d^ 0.63	–
Hypoglycemia events	22.4^c^ –	247 (3.4)	4.9 < 0.001^a^	56.0^d^ 0.20	54 (2) 0.95

^a^Superiority test

^b^Average across all age ranges

^c^Severe hypoglycaemia as defined by ADA

^d^Number of participants per 1000 patient-years

### Primary MACE composite endpoint

The primary MACE of the recently completed studies comprised similar elements: CV death, MI and stroke. The ACE study additionally included UA and HF into the primary composite endpoint.

In the DEVOTE study, Insulin degludec was non-inferior to Insulin glargine. Primary MACE occurred in 8.5% of the degludec group vs. 9.3% of the glargine group (HR 0.91; 95% CI 0.78–1.06; p < 0.001 for non-inferiority [10]). Participants of the CANVAS program had a reduced risk of cardiovascular events when using canagliflozin in comparison to placebo. 26.9 vs. 31.5 participants per 1000 patient-years had an event comprised in the primary MACE (HR 0.86; 95% CI 0.75–0.97; p < 0.001 for non-inferiority; p = 0.02 for superiority [21]). In the EXSCEL study the primary composite outcome occurred in 839 of 7356 patients in the once-weekly exenatide group compared to 905 of 7396 patients in the placebo group (HR 0.91; 95% CI 0.83–1.00). Thus, once-weekly exenatide was non-inferior with respect to safety but not superior with respect to efficacy (p < 0.001 for non-inferiority; p = 0.06 for superiority [24]). A total of 470 of 3272 (14.4%) participants of the acarbose group in the ACE trial had a primary outcome event compared to 479 of 3250 (14.7%) in the placebo group showing no reduction of CV risk (HR 0.98; 95% CI 0.86–1.11; p = 0.73 [23]).

### All-cause mortality

All-cause mortality was included in the secondary outcomes of DEVOTE and observed in 202 (5.3%) participants of the degludec group in comparison to 221 (5.8%) participants in the glargine group (HR 0.91; 95% CI 0.76–1.11; p = 0.35), revealing no significant between group difference [10]. Treatment with canagliflozin did not result in a decrease of all-cause mortality, showing no superiority to placebo in the first secondary outcome of the CANVAS program (17.3 vs. 19.5 HR 0.87; 95% CI 0.74–1.01; p = 0.24 [21]). In the EXSCEL trial all-cause mortality, a predefined secondary outcome, was 6.9% (n = 507) in the once-weekly exenatide vs. 7.9% (n = 584) in the placebo groups, respectively (HR 0.86; 95% CI 0.77–0.97 [24]. The ACE study could not demonstrate a significant difference between the treatment with acarbose or placebo with regard to all-cause mortality (216 (7%) of 3272 vs. 219 (7%) of 3250; HR 0.98; 95% CI 0.81–1.19; p = 0.85 [23]).

### Cardiovascular death

There was no significant difference in the occurrence of CV death comparing the two study groups confirming non-inferiority of Insulin degludec towards Insulin glargine (3.6% vs. 3.7%; HR 0.96; 95% CI 0.76–1.21; p = 0.71 [10]). Participants treated with canagliflozin less often died from CV causes, but the difference was not significant (11.6 vs. 12.8 participants per 1000 patient-years; HR 0.87; 95% CI 0.72–1.06; p = 0.94 [21]). In the EXSCEL trial 340 (4.6%) patients died of CV causes in the once-weekly exenatide group whereas 383 (5.2%) patients in the placebo group died as a result of CV events, showing no between group difference (HR 0.88; 95% CI 0.76–1.02 [24]). The rate of CV related deaths in the ACE study was similar to EXSCEL. 4.4% of patients died due to CV causes in the acarbose group and 5.0% in the placebo group. This between group difference was not considered to be significant (HR 0.89; 95% CI 0.71–1.11; p = 0.29 [23]).

### Fatal and/or non-fatal MI

Regarding the second component of the primary outcome, non-fatal MI, non-inferiority of Insulin degludec to Insulin glargine was confirmed (3.8% vs. 4.4%; HR 0.85; 95% CI 0.68–1.06; p = 0.15 [10]). In the CANVAS program a decrease in fatal or non-fatal MI could be observed in the canagliflozin group compared to placebo, but again no statistical significance was determined (11.2 vs. 12.6 participants per 1000 patient-years; HR 0.87, 95% CI 0.73–1.09 [21]). EXSCEL and ACE investigated fatal or non-fatal MI as a secondary outcome. In the once-weekly exenatide group 483 patients (6.6%) and 493 patients (6.7%) in the placebo group had a fatal or non-fatal MI showing no difference between the two treatment groups (HR 0.97; 95% CI 0.85–1.10 [24]). In ACE the numbers of fatal or non-fatal MI were 122 (3.7%) vs. 108 (3.3%), indicating no significant difference in the treatment with acarbose and placebo (HR 1.12; 95% CI 0.87–1.46; p = 0.38 [23]).

### Fatal and/or non-fatal stroke

Non-inferiority was confirmed for Insulin degludec regarding the primary endpoint non-fatal stroke. 1.9% of patients in the degludec and 2.1% of patients in the glargine group had a non-fatal stroke (HR 0.90; 95% CI 0.65–1.23; p = 0.50 [10]). As observed for CV death and fatal or non-fatal MI, fatal and non-fatal stroke occurrences decreased with canagliflozin without showing significant differences to the placebo group (7.9 vs. 9.6 participants per 1000 patient-years; HR 0.87; 95% CI 0.69–1.09 [21]). 187 patients (2.5%) had a fatal or non-fatal stroke being treated once-weekly with exenatide in comparison to 218 patients (2.9%) being given placebo. The number of events was not statistically different between the two groups (HR 0.85; 95% CI 0.70–1.03 [24]). Similarly, treatment with acarbose did not reveal a reduction of stroke events compared to placebo (2.3% vs. 2.4%; HR 0.97; 95% CI, 0.70–1.33; p = 0.83 [23]).

### Hospitalization for UA

In the DEVOTE study, UA leading to hospitalization was included into the extended primary endpoint. In both groups, degludec and glargine, 1.9% of patients were hospitalized due to UA (HR 0.95; 95% CI 0.68–1.31 [10]). The CANVAS program and EXSCEL did not analyse UA [21, 24]. In the ACE study hospital admission for UA was included in the five point MACE and independently investigated as a secondary outcome. 174 participants (5.3%) were hospitalized for UA after being treated with acarbose and 170 participants of the placebo group (5.2%; HR 1.02; 95% CI 0.82–1.26; p = 0.87) revealing no different outcomes [23].

### Hospitalization for HF

The DEVOTE study did not provide information on hospitalization for HF, whereas the CANVAS program confirmed superiority of canagliflozin to placebo with 5.5 vs. 8.7 participants per 1000 patient-years (HR 0.67; 95% CI 0.52–0.87 [21]). EXSCEL did not show a significant difference between the two treatment groups concerning hospitalization for HF: 219 patients (3.0%) had an event when treated with once-weekly exenatide and 231 patients (3.1%) when given placebo (HR 0.94; 95% CI 0.78–1.13 [24]). In ACE, 65 participants (2.0%) of the acarbose group were hospitalized for HF and 73 participants (2.2%) of the placebo group. This difference was also not significant (HR 0.89; 95% CI 0.63–1.24; p = 0.48 [23]).

### Renal events and/or microvascular effects

Adverse events associated with renal and urinary disorders were comparable between participants in the degludec and the glargine group in the DEVOTE study (3.8% vs. 4.5%) Acute kidney injury occurred in 1.8% of participants treated with Insulin degludec and in 2.5% of participants treated with Insulin glargine [10]. As the CANVAS program includes CANVAS-R, renal outcomes were specifically analysed. Progression of albuminuria was defined as primary outcome, whereas Albuminuria regression was defined as secondary outcome. Albuminuria progression was less frequent in participants assigned to canagliflozin than placebo (89.4 vs. 128.7 participants with an event per 1000 patient-years; HR 0.73; 95% CI 0.67–0.79). Regression of Albuminuria occurred more frequently in canagliflozin group than in the placebo group (293 vs. 188 participants with an event per 1000 patient-years; HR 1.70; 95% CI 1.51–1.91) and renal-related adverse events were observed with an event rate of 20 vs. 17 per 1000 patient-years (p = 0.32 [21]). EXSCEL classified renal events as “end stage renal failure needing chronic peritoneal/haemodialysis (including creation of fistula or other vascular access for haemodialysis) or renal transplantation”. 55 of 7344 participants of the once-weekly exenatide group and 65 of 7389 participants of the placebo group had a renal event as classified above (0.7% vs. 0.9% [24]). In the ACE trial the incidence of impaired renal function was not different between the acarbose and the placebo groups (rate ratio 0.81; 95% CI 0.54–1.23; p = 0.33 [23]).

### Pancreatic effects

Pancreatic effects were not analysed in the DEVOTE and ACE studies [10, 23]. In CANVAS there was no difference between canagliflozin and placebo regarding acute pancreatitis (0.5 vs. 0.4 events per 1000 patient-years, p 0.63 [21]). The number of patients with an adverse pancreatic event were 26 in the once-weekly exenatide and 22 in the placebo group of the EXSCEL trial (0.4% vs. 0.3% [24]).

### Serious hypoglycaemic events

The mean number of severe hypoglycaemic episodes as well as the number of nocturnal severe hypoglycaemic events was significantly reduced in participants in the Insulin degludec group compared to the Insulin glargine group (4.9% vs. 6.6% of patients with severe hypoglycaemia; rate ratio 0.73; 95% CI 0.60–0.89; p < 0.001 for superiority; 1.0% vs. 1.9% of patients with nocturnal severe hypoglycaemia; rate ration 0.47; 95% CI 0.31–0.73; p < 0.001 [10]). Participants of the CANVAS program had no higher risk of hypoglycaemia with canagliflozin than with placebo (50.0 vs. 46.4 events per 1000 patient-years; p = 0.20 [21]). This was also the case in participants of the EXSCEL trial: there was no difference in the rates of severe hypoglycaemia, neither when measuring only the first event (1.0 events per 100 patient-years in the once-weekly exenatide group and 0.9 events per 100 patient-years in the placebo group) nor when including recurrent events (1.6 events per 100 patient-years and 1.8 events per 100 patient-years [24]). There was also no between group difference in the rate of severe hypoglycaemic episodes in the ACE study (54 of 3272 (2%) vs. 52 of 3250 (2%) [23]).

### Amputations

The number of amputations, although relatively infrequent, was significantly increased in the canagliflozin group compared to the placebo group. 6.3 vs. 3.4 participants per 1000 patient-years had an amputation primarily of a lower limb (HR 1.97, 95% CI 1.41–2.75). Subgroup analysis revealed, that the higher risk was associated with a history of amputations or peripheral vascular disease and that the relative risk was comparable between subgroups [21]. In the DEVOTE and ACE studies, amputations were not listed as adverse events [10, 23]. For the treatment with once-weekly exenatide a non-traumatic amputation was reported for 128 of 7344 participants (1.7%). Similarly, 127 of 7389 participants (1.7%) of the placebo group had to undergo non-traumatic amputation [24].

In addition to the CVOTs that were already completed this year we would like point out one other study that will give further insight into the CV effects of albiglutide, another GLP-1 RA. The albiglutide trial is estimated to be completed in 2018 and will compare the effect of 30 mg albiglutide, 50 mg albiglutide and placebo on CVOs in T2DM patients with CVD.

## Discussion

The new trials gave insights into the CV effects of the basal insulin degludec, the SGLT-2 inhibitor canagliflozin, the GLP-1 RA once-weekly exenatide and the α-glucosidase inhibitor acarbose. Insulin degludec was compared to Insulin glargine U100. Since 2015 the new longer-acting Insulin glargine U300 has been approved for the European market, after initiation of DEVOTE. The DEVOTE study could confirm non-inferiority in terms of CV events. With regard to hypoglycaemic risks Insulin degludec was superior to Insulin glargine, both in rates of severe and nocturnal severe hypoglycaemia. Glycaemic control did not differ between the two groups. Also adverse events did not occur in different rates comparing Insulin degludec and Insulin glargine. Interestingly, it was reported that fasting glucose variability during the study was associated with higher risk of hypoglycaemia and of total mortality [25]. This observation is of interest because glucose variability has been suggested as an independent risk factor for diabetic complications [26]. Long lasting insulins provide patients with the possibility to consistently lower glucose levels. The more stable pharmacodynamics result in a half-life of more than 24 h and thereby reduce the risks of hypoglycaemia [27]. The DEVOTE study is only the second completed trial investigating CV effects of standard care medication like insulin, metformin or sulfonylurea. The ORIGIN trial compared CV outcomes of Insulin glargine with standard care and did not observe any differences between the two groups [9].

The CANVAS program displays a new approach, combining two studies after trial initiation. The combination of CANVAS and CANVAS-R increases participant numbers and statistical power. Additionally it enables the parallel analysis of separate primary objectives: CV outcomes and kidney disease progression. The combined CANVAS programs objective, superiority of canagliflozin towards placebo was achieved. Even though the primary endpoint was met with significant differences, the three components of the primary MACE endpoint—CV death, MI and stroke—only revealed minor benefits. In contrast to the separate primary outcomes, secondary outcomes like hospitalization for HF showed superiority for canagliflozin. Comparing the primary outcomes on CV risk of the SGLT-2 inhibitors canagliflozin and empagliflozin both showed superiority over placebo. Both agents had a positive effect on the progression of kidney disease [18, 21, 28]. The EMPA-REG OUTCOME study observed a significant risk reduction of microvascular outcome events which was mainly driven by a lower progression rate of kidney disease [28]. This possible benefit of SGLT-2 inhibitors on the progression rate of kidney disease was also observed in the CANVAS program [21]. In contrast to empagliflozin, canagliflozin appeared to increase the rate of bone fractures (15.4 vs. 11.9 participants with fracture per 1000 patient-years; HR 1.26; 95% CI 1.04–1.52 [21, 29] and amputations, prompting FDA and EMA to publish safety warnings [30–33]. The currently ongoing DECLARE-TIMI study will provide information on safety issues of dapagliflozin, enabling comparison with further SGLT-2 inhibitors. The contemporary analysis of real-world clinical practice CV-REAL included data from health records from six countries of patients that were newly started on SGLT-2 inhibitors and compared them with patients newly started on other glucose-lowering drugs. The SGLT-2 inhibitors varied according to countries included (canagliflozin, empagliflozin and dapagliflozin). This epidemiological study showed that the analysed SGLT-2 inhibitors can—in a real-world setting—reduce the risk of hospitalization for HF and of all-cause mortality by 39% and 51%, respectively, when compared with other glucose-lowering drugs. This study provides complementary information on clinical trials of SGLT-2 inhibitors, namely EMPA-REG OUTCOME and the CANVAS program [34]. In total, the results concerning CVOs of the CANVAS program are comparable to those published in previous studies for empagliflozin [18, 35].

EXSCEL adds information on glucose-lowering agents of the GLP-1 RA class to the already published ELIXA, LEADER and SUSTAIN-6 results. Once-weekly exenatide confirmed non-inferiority to placebo in regard of CV safety but was not seen as superior in regard of efficacy. This is comparable to lixisenatide in the ELIXA trial [15, 24]. Nevertheless once-weekly exenatide decreased all-cause mortality by 14%, a similar reduction as observed for liraglutide (15% risk reduction of all-cause death), even though, by formal reasons, due to the lack of significant impact on the primary composite endpoint, could not in a hierarchical statistical analysis be accepted as formally significant [16, 24]. No safety concerns were risen by any adverse events observed in the EXSCEL trial. A fifth GLP-1 RA, dulaglutide is currently tested for CV safety in the REWIND study, which is estimated to be completed in July 2018.

The ACE trial differed from the other presented CVOTs with regard to trial population. Pre-existing diabetes was an exclusion criterion and for inclusion participants required a history of CVD and impaired glucose tolerance. As this trial was conducted in China, trial population consisted to 97% of Han Chinese. It was shown, that acarbose was more effective in individuals consuming “Eastern” diets compared to “Western” diets. This might be a reason for the high number of prescriptions of acarbose in China, where it is the most common oral glucose-lowering medication. It is also prescribed as preventative medication for individuals with impaired glucose tolerance (summarized in [36]). Designed as a CVOT with a three point MACE the ACE trial could not achieve enough events, resulting in an updated five point MACE (adding hospitalization for HF and UA) as primary outcome. The primary endpoint was not reduced comparing acarbose with placebo. Also most secondary outcomes did not show a difference between the two treatment groups. The number of participants which developed diabetes during the follow-up of the ACE study was reduced by 18% in the acarbose group compared to the placebo group. This risk reduction of incident diabetes in the high CV risk population supports the fact, that acarbose is frequently prescribed as prevention for individuals with impaired glucose tolerance [23]. Anyhow, it is worthy of interest that postprandial hyperglycaemia was not significantly different between the two groups in the last 3 years of the study [23].

The recent CVOT results summarized in this overview indicate, that the utilization of Insulin degludec as glucose-lowering medication is safe in regard to CVOs and adverse events. Even though canagliflozin could also confirm its CV safety a warning of FDA and EMA for adverse effects like amputations and fractures should be kept in mind [30, 31, 33]. Once-weekly exenatide demonstrated safety with regard to CVOs and adverse events. ACE could confirm, that acarbose can be used without safety concerns.

One major strength of the CANVAS program is that it is, so far, one of the longest CVOTs initiated after 2008. All four newly published CVOTs had large numbers of participants with high CV risk. Still, the limitations of CVOTs remain patient selection criteria, trial duration, short follow-up time, lack of head-to-head comparison with standard care as discussed previously [8]. Comparisons between different trials is difficult due to varying inclusion criteria, baseline characteristics and trial durations and this needs to be acknowledged when interpreting the outcome of such comparisons.

The “ESC Guidelines for the diagnosis and treatment of acute and chronic heart failure” commented that in patients with HF the treatment of choice should be metformin, especially because no CV data were available for DPP-4 inhibitors and GLP-1 RAs. The positive results on CV safety seen for empagliflozin were recognized in the ESC guidelines, and they gave a reminder, that no class effect should be subscribed to SGLT-2 inhibitors [37]. After the publication of these guidelines Rydén and colleagues expressed certain concerns on these suggestions and presented an update on ESC HF guidelines: “DPP-4 inhibitors (gliptins) increase plasma levels of incretins by inhibiting their breakdown, thereby augmenting insulin release. The drugs do not have any effect on cardiovascular events and, apart from the observations made with saxagliptin and alogliptin, there is no compelling evidence that this class of drugs affects heart failure. By contrast, long-acting GLP-1 receptor agonists act as incretin mimetics, improve glycaemic indices, and either have no effect on (lixisenatide) or seem to reduce (liraglutide and semaglutide) cardiovascular events, with no effect on hospital admissions for heart failure” [38].

Efforts are made to provide CVO information on the standard glucose-lowering agents insulin, metformin and sulfonylurea. A meta-analysis concluded, that a CV risk of metformin in T2DM patients could not be determined mainly due to the low amount of information currently available [39]. The REMOVAL trial recently investigated the influence of metformin on atherosclerosis progression in long-standing type 1 diabetes mellitus (T1DM) patients with high CV risk. Adding metformin to the insulin therapy and standard of care did not significantly affect atherosclerosis progression. However, metformin treatment had a positive effect on HbA1c, body weight and LDL-cholesterol [40, 41]. In addition to REMOVAL, the EMERALD study currently investigates the influence of metformin on CV function in adolescent T1DM patients and is bound to be completed in 2018 (NCT01808690).

Sulfonylureas have so far also not been analysed stand-alone regarding CV effects. The “Cardiovascular Outcomes in Participants with Type 2 Diabetes Mellitus” study, which compares CV outcomes of several drug classes (SGLT-2 inhibitors, GLP-1 RAs, DPP-4 inhibitors, Thiazolidinedione, Sulfonylureas and Insulin) could give further insight into this question. This study is estimated to be completed by the end of 2017 (NCT03249506). Nevertheless, studies like the currently published TOSCA-IT and the still running CAROLINA provide a head-to-head comparison of sulfonylureas with pioglitazone and the DDP-4 inhibitor linagliptin [42].

The large-scale study TOSCA-IT was initiated to compare the efficacy of pioglitazone, a PPAR-γ agonist, and sulfonylurea as add-ons to metformin on CV outcomes. 3028 participants with inadequately controlled metformin monotherapy were randomized. The trial was designed as a Prospective Randomised Open Blinded Evaluation (PROBE) study, i.e. the event adjudicators were unaware of treatment assignment. The primary outcome was a composite of all-cause death, non-fatal MI, non-fatal stroke or urgent coronary revascularization. No significant differences between the two treatment groups—pioglitazone vs. sulfonylurea—could be observed in regard of the primary and secondary outcomes. The number of hypoglycaemic events was significantly lower and LDL-cholesterol levels higher in the pioglitazone than in the sulfonylurea group. These results suggest, that both drugs are suitable options as add-on treatment to metformin, with benefits for hypoglycaemia and LDL-cholesterol when using pioglitazone [43]. A previous CVOT with pioglitazone (PROactive) described a significant reduction of non-fatal MI and stroke in patients with T2DM and a high risk of macrovascular events. However, other end-points like heart failure showed a drastic increase in the pioglitazone group [44]. These results could not be confirmed by the TOSCA-IT study. Vaccaro and colleagues suggest, that these discrepancies arouse by the different outcomes assessed, the study populations and the choice of comparator. The low risk population of TOSCA-IT (only 11% with CVD) could mask minor benefits of pioglitazone. Indeed, post hoc on-treatment results are in agreement with previous findings [43, 44].

Not only glucose-lowering medications are tested for CV safety. The therapeutic monoclonal antibody canakinumab targets interleukin-1β and is used as anti-inflammatory medication. Several observations suggest that a specific targeting of interleukin-1β could be used as secondary prevention of atherosclerotic events. Therefore CANTOS investigated the occurrence of non-fatal MI, non-fatal stoke or CV death as primary composite endpoint after the treatment with canakinumab. 40% of participants had a history of diabetes. Canakinumab could dose-dependently reduce the risk of CV events (primary and secondary endpoints) compared to placebo. All-cause mortality was comparable between the treatment groups, but patients of the canakinumab group died significantly more often due to infection or sepsis. These patients that died were more likely to have diabetes than those who did not die from infection [45].

Several additional trials ranging the most common glucose-lowering medications will be completed in the next 2 years. CAROLINA (estimated end date 03.2019) and CARMELINA (estimated end date 12.2017) will present information on the DPP-4 inhibitor linagliptin. The first CVOTs on DPP-4 inhibitors (SAVIOR-TIMI53, EXAMINE and TECOS) reported neutral effects on the composite MACE endpoint. SAVIOR-TIMI53 and EXAMINE however indicated an increase in risk of hospitalization for HF [12, 13]. Three agents of the GLP-1 RA class have already been successfully tested for CV safety (liraglutide, lixisenatide and semaglutide [16, 17, 44]). Also once-weekly exenatide is safe with regard to CVOs. Two currently ongoing studies will present the results for further agents in the next year: REWIND (dulaglutide) and the albiglutide trial (albiglutide). SGLT-2 inhibitors are a relatively new class of glucose-lowering drugs. Therefore, several trials on CVO are currently running and will be completed in 2019 (DECLARE-TIMI—dapagliflozin and VERTIS—ertugliflozin) to add new information on further agents to the results obtained in the EMPA-REG OUTCOME study and the CANVAS program. With regard of the new CVOT results, empagliflozin, liraglutide and semaglutide remain the preferred second- and third-line medication in patients with T2DM adding canagliflozin as an additional option [46].

## Conclusion

Important CVOTs assessing CV safety of glucose-lowering medication were completed and presented in 2017. They reached their primary outcomes and confirmed previous studies that indicated no increased CV risk of glucose-lowering drugs. Insulin degludec showed non-inferiority to Insulin glargine (DEVOTE). The CANVAS program demonstrated superiority of canagliflozin to placebo in the primary endpoint. Once-weekly exenatide (EXSCEL) and acarbose (ACE) were non-inferior to placebo and no safety concerns were raised by the presented results. Many additional CVOTs are estimated to be completed within the next 2 years and will provide additional insights into CV safety of glucose-lowering drugs.

## Abbreviations

D&CVD: diabetes & cardiovascular disease
EASD: European Association for the Study of Diabetes
CV: cardiovascular
CVD: cardiovascular disease
FDA: Food and Drug Administration
EMA: European medicine agency
HR: hazard ration
CI: confidence interval
CVOT: cardiovascular outcome trial
DPP-4: dipeptidyl-peptidase-4
SGLT-2: sodium-glucose cotransporter-2
GLP-1: glucagon-like-peptide-1
RA: receptor agonist
T2DM: type 2 diabetes mellitus
MACE: major adverse cardiovascular event
MI: myocardial infarction
UA: unstable angina
HF: heart failure
T1DM: type 1 diabetes mellitus
LDL: low density lipoprotein
ESC: European Society of Cardiology
PPAR: peroxisome proliferator-activated receptor
ACS: acute coronary syndrome
AHA: anti-hyperglycaemic agent
ACEI: angiotensin-converting-enzyme inhibitor
ARB: angiotensin-receptor blocker
ADA: American Diabetes Association
CVO: Cardiovascular outcome
