# Correction to: Updates on cardiovascular outcome trials in diabetes

**DOI:** 10.1186/s12933-017-0633-4

**Published:** 2017-11-15

**Authors:** Oliver Schnell, Lars Rydén, Eberhard Standl, Antonio Ceriello, on behalf of the D&CVD EASD Study Group

**Affiliations:** 1 Forschergruppe Diabetes e. V., Ingolstaedter Landstraße 1, Neuherberg, 85764 Munich, Germany; 2 Cardiology Unit, Department of Medicine K2, Karolinska Institute, 171 76 Stockholm, Sweden; 3 Institut d’Investigacions Biomèdiques August Pi i Sunyer (IDIBAPS) and Centro de Investigación, Biomedica en Red de Diabetes y Enfermedades Metabólicas Asociadas (CIBERDEM), Barcelona, Spain; 4 Department of Cardiovascular and Metabolic Diseases, IRCCS Multimedica Sesto San Giovanni, Via Milanese 300, 20099 Milan, Italy

## Correction to: Cardiovasc Diabetol (2017) 16:128 10.1186/s12933-017-0610-y

Following publication of the original article [1], the authors submitted a corrected version of Table 4 (see below), as the original version contained a mistake.

**Table 4:** Comparison of outcome results from terminated CVOTs in comparison to placebo

	SAVOR-TIMI53 [1, 2]		EXAMINE [3, 4]		TECOS [5]		ELIXA [6]		EMPA-REG OUTCOME [7]		LEADER [8]	
	Class	HR (95% CI)	Class	HR (95% CI)	Class	HR (95% CI)	Class	HR (95% CI)	Class	HR (95% CI)	Class	HR (95% CI)
		p value		p value		p value		p value		p value		p value
Cardiovascular endpoints												
Primary composite MACE	CV death, MI, or stroke	1.00 (0.89–1.12) 0.99	CV death, MI, or stroke	0.96 (≤ 1.16) 0.32	CV death, MI, UA, or stroke	0.98 (0.89–1.08) 0.65	CV death, MI, UA, or stroke	1.02 (0.89–1.17) 0.81	CV death, MI, or stroke	0.86 (0.74–0.99) 0.04^a^	CV death, MI, or stroke	0.87 (0.78–0.97) 0.01
Cardiovascular death	Primary endpoint	1.03 (0.87–1.22) 0.72	Primary endpoint	0.79 (0.60–1.04) 0.10	Secondary endpoint	1.03 (0.89–1.19) 0.71	Primary endpoint	0.98 (0.78–1.22) 0.85	Primary endpoint	0.62 (0.49–0.77) < 0.001	Primary endpoint	0.78 (0.66–0.93) 0.007
Myocardial infarction	Primary endpoint	0.95 (0.80–1.12) 0.52	Primary endpoint	1.08 (0.88–1.33) 0.47	Secondary endpoint	0.95 (0.81–1.11) 0.49	Primary endpoint	1.03 (0.87–1.22) 0.71	Primary endpoint	0.87 (0.70–1.09) 0.23	Primary endpoint	0.86 (0.73–1.00) 0.046
Stroke	Primary endpoint	1.11 (0.88–1.39) 0.38	Primary endpoint	0.91 (0.55–1.50) 0.71	Secondary endpoint	0.97 (0.79–1.19) 0.76	Primary endpoint	1.12 (0.79–1.58) 0.54	Primary endpoint	1.18 (0.89–1.56) 0.26	Primary endpoint	0.86 (0.71–1.06) 0.16
Hospitalization for unstable angina	Secondary endpoint	1.19 (0.89–1.60) 0.24	Secondary endpoint	0.90 (0.60–1.37) 0.632	Secondary endpoint	0.90 (0.70–1.16) 0.42	Primary endpoint	1.11 (0.47–2.62) 0.81	Secondary endpoint	0.99 (0.74–1.34) 0.97	Extended primary endpoint	0.98 (0.76–1.26) 0.87
Hospitalization for heart failure	Secondary endpoint	1.27 (1.07–1.51) 0.007	Extended primary endpoint	1.19 (0.90–1.58) 0.220	Secondary endpoint	1.00 (0.83–1.20) 0.98	Secondary endpoint	0.96 (0.75–1.23) 0.75	Secondary endpoint	0.65 (0.50–0.85) 0.002	Extended primary endpoint	0.87 (0.73–1.05) 0.14

	Event rate (%) active group		Event rate (%) active group		Event rate (%) active group		Event rate (%) active group		Event rate (%) active group		Event rate (%) active group	
Primary composite MACE	7.3		11.3		9.6		13.4		10.5		13.0	

	No. (%) p value		No. (%) p value		No. (%) p value		No. (%) p value		No. (%) p value		No. (%) p value	
Non-cardiovascular endpoints												
Renal event	5.8 0.04		0.9 0.88		1.4 –		1.6 –		5.2 –		5.7 0.003	
Acute pancreatitis	0.3 0.77		0.4 0.5		0.3 0.07		0.2 –		0.3^b^ –		0.4 0.44	
Hypoglycemia events	15.3 < 0.001		6.7 0.74		2.2 0.33		16.6 0.14		2.8		2.4 0.02	

	SUSTAIN 6 [9]		EXSCEL [10]		DEVOTE [11]		CANVAS program [12]		ACE [13]	
	Class	HR (95% CI) p value	Class	HR (95% CI) p value	Class	HR (95% CI) p value	Class	HR (95% CI) p value	Class	HR (95% CI) p value
Cardiovascular endpoints										
Primary composite MACE	CV death, MI, or stroke	0.74 (0.58–0.95) 0.02^a^	CV death, MI or stroke	0.91 (0.83–1.00) 0.06^a^	CV death, MI or stroke	0.91 (0.78–1.06) < 0.001	CV death, MI or stroke	0.86 (0.75–0.97) 0.02^a^	CV death, MI, stroke, UA and HF	0.98 (0.86–1.11) 0.73
Cardiovascular death	Primary endpoint	0.98 (0.65–1.48) 0.92	Secondary endpoint	0.88 (0.76–1.02)	Primary endpoint	0.96 (0.76–1.21) 0.71	Primary endpoint	0.87 (0.72–1.06)	Secondary endpoint	0.89 (0.71–1.11) 0.29
Myocardial infarction	Primary endpoint	0.74 (0.51–1.08) 0.12	Secondary endpoint	0.97 (0.85–1.10)	Primary endpoint	0.85 (0.68–1.06) 0.15	Primary endpoint	0.89 (0.73–1.09)	Secondary endpoint	1.12 (0.87–1.46) 0.38
Stroke	Primary endpoint	0.61 (0.38–0.99) 0.04	Secondary endpoint	0.85 (0.70–1.03)	Primary endpoint	0.90 (0.65–1.23) 0.50	Primary endpoint	0.87 (0.69–1.09)	Secondary endpoint	0.97 (0.70–1.33) 0.83
Hospitalization for unstable angina	Extended primary endpoint	0.82 (0.47–1.44) 0.49	–	–	Primary endpoint	0.95 (0.68–1.31) 0.74	–	–	Secondary endpoint	1.02 (0.82–1.26) 0.87
Hospitalization for heart failure	Extended primary endpoint	1.11 (0.77–1.61) 0.57	Secondary endpoint	0.94 (0.78–1.13)	–	–	Secondary endpoint	0.67 (0.52–0.87)	Secondary endpoint	0.89 (0.63–1.24) 0.48

	Event rate (%) active group	Event rate (%) active group	Event rate (%) active group	Event rate (%) active group	Event rate (%) active group
Primary composite MACE	6.6	11.4	8.5	26.9^d^	14.4

	No. (%) p value	No. (%) p value	No. (%) p value	No. (%) p value	No. (%) p value
Non-cardiovascular endpoints					
Renal event	3.9 –	55 (0.7)	3.8 –	19.7^d^ 0.32	–
Acute pancreatitis	0.55 –	26 (0.4)	– –	0.5^d^ 0.63	–
Hypoglycemia events	22.4^c^ –	247 (3.4)	4.9 < 0.001^a^	56.0^d^ 0.20	54 (2) 0.95

^a^Superiority test

^b^Average across all age ranges

^c^Severe hypoglycaemia as defined by ADA

^d^Number of participants per 1000 patient-years

The original article has been corrected.

**References**

1. Leiter LA, et al. Efficacy and safety of saxagliptin in older participants in the SAVOR-TIMI 53 trial. Diabetes Care. 2015;38(6):1145–53.
2. Scirica BM, et al. Saxagliptin and cardiovascular outcomes in patients with type 2 diabetes mellitus. N Engl J Med. 2013;369(14):1317–26.
3. White WB, et al. Alogliptin after acute coronary syndrome in patients with type 2 diabetes. N Engl J Med. 2013;369(14):1327–35.
4. Zannad F, et al. Heart failure and mortality outcomes in patients with type 2 diabetes taking alogliptin versus placebo in EXAMINE: a multicentre, randomised, double-blind trial. Lancet. 2015;385(9982):2067–76.
5. Green JB, et al. Effect of sitagliptin on cardiovascular outcomes in Type 2 diabetes. N Engl J Med. 2015;373(3):232–42.
6. Pfeffer MA, et al. Lixisenatide in patients with Type 2 diabetes and acute coronary syndrome. N Engl J Med. 2015;373(23):2247–57.
7. Zinman B, et al. Empagliflozin, cardiovascular outcomes, and mortality in Type 2 diabetes. N Engl J Med. 2015;373(22):2117–28.
8. Marso SP, et al. Liraglutide and cardiovascular outcomes in Type 2 diabetes. N Engl J Med. 2016;375(4):311–22
9. Marso SP, et al. Semaglutide and cardiovascular outcomes in patients with Type 2 diabetes. N Engl J Med. 2016;375(19):1834–44.
10. Holman RR, et al. Effects of once-weekly exenatide on cardiovascular outcomes in Type 2 diabetes. N Engl J Med. 2017;377(13):1228–39.
11. Marso SP, et al. Efficacy and safety of degludec versus glargine in Type 2 diabetes. N Engl J Med. 2017;377(8):723–32.
12. Neal B, et al. Canagliflozin and cardiovascular and renal events in Type 2 diabetes. N Engl J Med. 2017;377(7):644-57.
13. Holman RR, et al. Effects of acarbose on cardiovascular and diabetes outcomes in patients with coronary heart disease and impaired glucose tolerance (ACE): a randomised, double-blind, placebo-controlled trial. Lancet Diabetes Endocrinol. 2017;S2213-8587(17):30309–1.
